# Medical Fees

**Published:** 1845-10-01

**Authors:** 


*Medical Fees.*—It was said by Dr. Johnson, that "every man has found in Physicians great liberality and dignity of sentiment, very prompt effusion of beneficence, and willingness to exert a lucrative art where there is no hope of lucre." This tribute to the philanthropic character of the profession, has, probably, not been forfeited since Johnson's day. No impartial observer can accuse physicians, in the aggregate, of being actuated by mercenary views, albeit there may be some inglorious exceptions to the general rule. But medical men, like all other men, must live; their families must be supported, and their children educated; and it is not an unreasonable desire on their part, to seek to make some provisions against casualties, and old age. Hence, it cannot but be expected that the subject of medical fees is one of interest and importance to them. If any apology were necessary for the discussion of this subject, it might be found in certain important relations to professional improvement, to which allusion will presently be made.

A physician must necessarily bestow a good proportion of his time and labor without any compensation therefor. A certain portion of every community require medical attendance without possessing the ability to make any remuneration. Sickness itself, frequently destroys the ability to reward the attention and skill which have either effected restorement, or soothed the sufferings incident to protracted, fatal disease. Of this we do not complain; it is our privilege, as well as duty, to render our services cheerfully in many cases without any expectation of pecuniary recompense. We would not cite this as a difficulty with which the profession has to contend. But there are other classes of patients, and the proportion which they bear in a physician's practice is generally not small, whose views and habits with regard to medical fees, are proper subjects of criticism and complaint. A certain portion of the community expect to obtain a physician's services gratuitously, when they are able to pay something, at least, if not a full compensation. They seem to act under the belief that *they* enjoy a peculiar immunity from any obligations to pay for medical attendance. They are apt to assume a very patronizing air toward the faculty, apparently thinking that it should be esteemed a privilege to serve them for nothing. They are particularly sensitive, if, when they apply to a physician, he is not exceedingly prompt and active in his attendance, and are very prone to think he is not sufficiently diligent and assiduous in his visits and attentions. If called upon with a *bill*, they think it hard for poor men like themselves to be expected to pay for what costs the physician little or nothing, viz: his time, and labor, and perhaps medicine. The truth is, they do not mean to pay, and if the physician presumes to persist in his solicitations, they extend their valuable patronage to some other member of the profession, being very careful ever afterward to evince their gratitude for the services of the predecessor by disparaging his abilities, or other malicious insinuations. Another class is a grade above that just mentioned. Persons of this class are very requiring, and not easily satisfied with the efforts of the Doctor while he is on duty. When the time for payment arrives, they are astonished at the amount of the bill: they cant pay so great a sum, and the bill, at length is accommodated, not to their ability, but to their disinclination to pay more than a small portion of its amount. Others expect to pay reasonable charges for medical services, but after a very unreasonable period. The Doctor can afford to wait, or if he cant afford it, he ought not to expect to do otherwise: all other bills take precedence; and, finally, this, after much solicitation, receives reluctant attention. In the mean time, however, the nature and extent of the services are forgotten, and the Doctor when he effects a settlement, is fortunate to escape with some crusty hints that Doctors have no consciences, that they live on society without labor, &c.—Another class, abundantly able to pay, not only require the physician to wait an indefinite period, but, in the end, think it incumbent on him to take something or rather any thing else than money, and that at a very exorbitant valuation, It is apparently a principle with them not to pay a Doctor's bill in cash, and if the Doctor does not need whatever it is convenient to substitute, he must be content to wait.

We might enumerate other classes, but as we are writing for the profession, it would be quite superfluous. The truth is, with some most honorable exceptions, medical fees in this country have not that respect and consideration which are attached to other pecuniary obligations. Many have no compunctions in forfeiting them entirely if practicable; others insist that the physician shall reduce his demands greatly below their just amount; the majority oblige him to wait until it is perfectly convenient to them to pay, and not a few contend that he has no claim to a payment in money. Now all this, to our mind, indicates a wrong condition of things. Either a physician is bound to labor for nothing, or he is not. Either his services have a certain value to be represented in standard currency, or they have not. If he be not bound to give his services, and if they do have any specific, pecuniary value, then his just demands should be held on a par, at least, with those of other men. We desire greatly to see an improvement in the present state of things, and we believe it to be in the power of the profession to effect it. There is no reason why medical business should not be considered a cash business, at least so far as this, that credit, if given, is by consent of both parties, not to be claimed as a matter of course, and regulated entirely by the patient. It must be the case that bills in many cases cannot be conveniently paid immediately after the service is rendered, but great advantages would result from having them collectible immediately, and be so considered in all cases where it is convenient to liquidate them. The following are some of the advantages which would accrue from this improvement.

The physician could perhaps afford to charge smaller fees than he can live by in the existing state of things. He would be able to do better justice to his own creditors, and those dependent on him for support. He would know more definitely the amount of his available income, and be able to regulate his expenses accordingly. But, more than all, he would be spared a vast amount of time and perplexity, which would conduce directly to his professional improvement and usefulness. In the place of having to bestow half his thoughts and exertions to devise where and how to collect funds enough to meet his own obligations, he would have the greater part of his leisure from active professional occupation to devote to study and scientific pursuits. and in these, as in his practical duties, he would have a clearer intellect, and a more buoyant spirit, than, we fear, most, under the present state of things, can lay claim to. We throw out these reflections for the consideration of our readers. By uniting in the necessity of a reform, and combining to take judicious means to effect it, we believe that the more sensible portion of community could be led to see its propriety, and to perceive, also, that *their* interests, not less than those of the profession, are involved in the subject. If the subject can be once fairly thrown upon its merits with the profession and the public, a reform would surely follow.

